# TCF12 enhances angiogenesis and affects sorafenib response in liver cancer via HIF-1α interaction

**DOI:** 10.17305/bb.2025.12022

**Published:** 2025-04-04

**Authors:** Yuanbin Chen, Xiaolong Wang, Jin Chen, Min Dai, Xinyue Zhang, Jie Yin, Xiao He

**Affiliations:** 1 Department of General Surgery, Affiliated Haian Hospital of Nantong University, Nantong, China; 2 Department of General Surgery, The Second People’s Hospital of Nantong, Nantong, China

**Keywords:** Transcription factor 12, TCF12, hypoxia-inducible factor 1-alpha, HIF-1α, angiogenesis, sorafenib

## Abstract

Transcription factor 12 (TCF12), a member of the basic Helix-Loop-Helix (bHLH) protein family, plays a crucial role in regulating cell growth and differentiation. It has been implicated in the development and progression of malignant tumors; however, its specific mechanisms in vascularization and drug resistance in liver cancer remain poorly understood. This study aims to explore how the interaction between TCF12 and hypoxia-inducible factor 1-alpha (HIF-1α) affects vascularization and drug sensitivity in liver cancer. Using bioinformatics analysis (*n* ═ 374 TCGA samples and *n* ═ 50 clinical specimens), we assessed TCF12 expression levels in liver cancer and evaluated their association with patient prognosis. Gene Set Enrichment Analysis (GSEA) was employed to identify related signaling pathways. The expression of TCF12 in liver cancer tissues was examined via Western blotting and immunohistochemistry, while Kaplan-Meier survival analysis was used to analyze the relationship between TCF12 expression and overall survival. Functional assays—including scratch wound repair, tube formation, and endothelial cell permeability tests—were conducted to assess TCF12’s role in angiogenesis. Cell viability assays were performed to evaluate the impact of TCF12 on sorafenib sensitivity, and co-immunoprecipitation experiments were carried out to investigate the interaction between TCF12 and HIF-1α. Our bioinformatics analysis revealed that both TCF12 and HIF-1α are significantly overexpressed in liver cancer and are associated with poor prognosis. Immunohistochemical staining showed a positive correlation between TCF12 expression and the vascularization marker CD31. Furthermore, survival analysis demonstrated that patients with elevated TCF12 expression had significantly shorter overall survival. Functional assays indicated that TCF12 knockdown suppressed blood vessel formation and reduced endothelial cell permeability. Moreover, reducing TCF12 expression increased the sensitivity of liver cancer cells to sorafenib. Notably, overexpression of HIF-1α reversed these effects, and co-immunoprecipitation experiments confirmed a direct interaction between TCF12 and HIF-1α. In summary, this study demonstrates that TCF12 is highly expressed in liver cancer and is associated with poor prognosis. TCF12 promotes angiogenesis by stabilizing HIF-1α and modulates tumor sensitivity to sorafenib, highlighting its potential as a therapeutic target in liver cancer.

## Introduction

In 2022, liver cancer remained the sixth most common cancer worldwide, with over 860,000 new cases and 750,000 deaths annually. Although its incidence rate is not the highest, the mortality rate is alarmingly high, reflecting the limited treatment options and generally poor prognosis associated with the disease. Liver cancer often develops insidiously and is typically diagnosed at an advanced stage. At this point, patients primarily receive interventional therapies and targeted drug treatments [1], with anti-angiogenic therapy playing a crucial role in prolonging survival. Vascular endothelial growth factor (VEGF), the most prominent pro-angiogenic factor, binds to the VEGF receptor (VEGFR) on endothelial cell (EC) membranes, triggering cell migration and increased permeability—processes that promote angiogenesis. However, the clinical efficacy of anti-vascular drugs targeting VEGF or VEGFR has been modest. Many patients develop resistance to treatment, leading to rapid disease progression. For example, sorafenib—a multi-target tyrosine kinase inhibitor that targets VEGFR—has shown some benefit in extending progression-free survival by approximately three months, but it has not significantly improved overall survival (OS). Moreover, only about 30% of patients with advanced liver cancer respond to sorafenib, and acquired resistance usually develops within six months [2, 3]. These challenges highlight the need for further investigation into the mechanisms of tumor angiogenesis to overcome resistance to anti-angiogenic therapies. Resistance to anti-angiogenic treatment is driven by complex mechanisms, including alterations in the tumor microenvironment, the upregulation of alternative pro-angiogenic factors, and epigenetic regulation. Notably, prolonged anti-angiogenic therapy induces intratumoral hypoxia, which triggers the secretion of additional pro-angiogenic factors and alters the behavior of ECs. This activation leads to morphological changes and loosening of intercellular junctions, increasing vascular permeability. As a result, tumor cells more easily invade the vasculature, facilitating metastasis [4]. This phenomenon—often referred to as the “incomplete” formation of blood vessels—represents a key resistance mechanism following anti-angiogenic therapy-induced hypoxia [5, 6]. It is well-established that anti-angiogenic therapy induces hypoxia, which in turn activates hypoxia-inducible factors (HIFs). HIF is a heterodimeric transcription factor composed of HIF-1α and HIF-1β. Under normoxic conditions, HIF-1α is ubiquitinated and degraded. In contrast, under hypoxic conditions, HIF-1α stabilizes, translocates to the nucleus, and binds to hypoxia response elements, promoting the transcription of pro-angiogenic factors, such as VEGF and angiopoietin-2. These factors drive EC activation, recruit endothelial progenitor cells, stimulate pericyte growth, and increase vascular permeability [7]. In colon cancer, for instance, HIF-1α induces the expression of Cyr61, another pro-angiogenic factor that contributes to increased vascular permeability [8]. HIF-1α’s stabilization under hypoxia is essential for its function as a transcriptional regulator. It contains a basic helix-loop-helix (bHLH) domain that allows it to form heterodimers with other HIF-1 family members, a process critical for transcriptional activity. Additionally, HIF-1α can interact with the histone acetyltransferase p300 to enhance the expression of downstream genes [9]. Recent studies have also shown that SIRT6 promotes the deubiquitination and stabilization of HIF-1α in both hypoxic and normoxic conditions [10].

Transcription factor 12 (TCF12) is a member of the bHLH protein family, which includes transcription factors characterized by two distinct motifs: the HLH domain and an adjacent basic region. These factors play essential roles in various cellular processes, including myogenesis, neurogenesis, and lymphocyte differentiation [11, 12]. The bHLH family is divided into two groups: class A proteins, which are broadly expressed, and class B proteins, which are tissue-specific [13]. Class A proteins, also known as E proteins, can bind directly to E-box sequences (CANNTG) in DNA and include TCF3, TCF4, and TCF12. As a member of this family, TCF12 regulates gene expression by forming dimers with other E proteins through its bHLH domain, thereby influencing cell growth, metastasis, differentiation, and other cellular functions. Notably, aberrant TCF12 expression is closely linked to tumor progression. In liver cancer, TCF12 promotes lumen formation in vascular ECs by upregulating CXCR4 expression, which in turn enhances tumor cell migration and invasion, contributing to disease advancement [14]. In colorectal cancer, TCF12 weakens intercellular adhesion and promotes cell migration by suppressing the expression of the tight junction protein E-cadherin [15, 16]. Furthermore, some studies suggest that TCF12 is regulated by HIF-1α, facilitating metastasis in rectal cancer [17]. These findings underscore the critical role of TCF12 in cancer development. This study aims to assess TCF12 expression levels in liver cancer using data from the TCGA database and Western blotting, and to analyze the relationship between TCF12 expression and patient prognosis. Additionally, we performed cell-based functional assays to examine the effects of TCF12 and HIF-1α on migration, tube formation, permeability, and other phenotypic characteristics of liver cancer ECs. We also evaluated how TCF12 and HIF-1α influence the sensitivity of these cells to the anticancer drug sorafenib. Finally, we explored the underlying mechanisms of interaction between TCF12 and HIF-1α.

## Materials and methods

### Datasets and patient samples

This study integrated RNA sequencing data with clinical information from the TCGA-LIHC dataset, which includes 374 liver cancer tissue samples and 50 normal liver tissue samples. Additionally, we collected 50 liver cancer tissue samples from Haian Hospital of Nantong University for immunohistochemical analysis, along with four paired samples of liver cancer and adjacent normal liver tissues for Western blot analysis. All clinical samples were obtained from patients with histologically confirmed hepatocellular carcinoma (HCC) who had not received prior radiotherapy or targeted therapy. Exclusion criteria included non-primary liver tumors and incomplete clinical records.

### Scratch test

Lentivirus-infected ECDHCC-1 cells were seeded in 6-well plates at a density of 3 × 10^5^ cells per well and cultured for 24 h until reaching approximately 80% confluence. The old culture medium was then discarded, and the cell monolayer was scratched using a yellow pipette tip. Cells were rinsed twice with PBS to remove any detached debris. Fresh ECM was added, and the cells were further cultured. Images were captured under a microscope both immediately after scratching and again after an additional 48 h of incubation. Wound closure was subsequently analyzed using ImageJ software.

### Angiogenesis experiments

Dissolve Matrigel overnight at 4 ^∘^C. Pre-cool the 96-well plate and pipette tips before use. While maintaining the temperature at 4 ^∘^C, add 50 µL of Matrigel to each well. Then, incubate the plate at 37 ^∘^C for 30 min to allow the gel to solidify. After incubation, seed each well with 4 × 10^4^ cells and incubate for 6 h. At the 8-h mark, observe tube formation under a microscope and analyze tube lumen differences using Image-Pro Plus 6.0 software.

### Immunohistochemical staining

Place the tissue chip in a dryer set to 80 ^∘^C for approximately 20 min. Next, immerse the tissue chip in two xylene baths, soaking for 15 min in each. Proceed by sequentially soaking the tissue chip in 100%, 90%, 80%, and 70% ethanol, for 3 min in each solution. Afterward, soak the tissue chip in ultrapure water for 5 min. Transfer the chip to an alkaline repair solution and heat it in a pressure cooker until steam is produced. Continue heating for an additional 3 min, then cool the tissue chip in ice water. Wash the chip three times with PBST. Using a histochemistry pen, outline the tissue along its edges. Incubate the tissue with a blocking agent at room temperature for 5 min, then apply the primary antibody and incubate overnight at 4 ^∘^C. The next day, allow the tissue chip to rewarm at room temperature for 30 min. Wash three times with PBST, then apply the secondary antibody and incubate at room temperature for 20 min. Wash again three times with PBST. Incubate the tissue chip with 1× DAB, stopping the reaction with tap water once a brown color appears. Stain with hematoxylin for 30 s, then rinse with tap water. Remove any remaining traces of the histochemistry pen. Dehydrate the tissue chip by soaking in 70% and 80% ethanol for 3 min each, followed by 90% and absolute ethanol for 5 min each. Then, immerse the chip twice in xylene for 8 min each. Finally, seal the slides with neutral resin, dry in a fume hood, and photograph under a microscope.

### Western blot

Begin by extracting cellular or tissue proteins and preparing the SDS-PAGE gel. Place the gel in the electrophoresis tank and add the electrophoresis buffer. Load protein samples and molecular weight markers into the appropriate wells. Set the voltage to 80 V to allow the proteins to migrate through the stacking gel, then increase the voltage to 120 V for separation. Once electrophoresis is complete, carefully remove the gel. Sequentially soak the PVDF membrane in methanol and then in double-distilled water (ddH_2_O). Assemble the transfer sandwich in the following order (from the positive to the negative electrode): sponge pad, filter paper, gel, PVDF membrane, filter paper, and sponge pad. Ensure the transfer setup is placed in an ice-filled trough to prevent overheating. Perform the protein transfer at 300 mA for 90 min. After transfer, block the PVDF membrane with milk at 4 ^∘^C for 2 h. Wash the membrane three times with Tris-buffered saline with Tween 20 (TBST) for 10 min each. Add the primary antibody and incubate the membrane overnight at 4 ^∘^C. The following day, allow the membrane to equilibrate at room temperature for 30 min, then wash three times with TBST for 10 min each. Apply the secondary antibody and incubate at room temperature for 30 min, followed by an additional 2-h incubation. Wash again three times with TBST for 10 min each. Finally, prepare the developing reagent, apply an appropriate amount to the PVDF membrane, and visualize the bands using a developing machine. Antibody dilutions: TCF12 (1:500 for IHC, 1:1000 for WB), HIF-1α (1:1000 for IHC/WB), CD31 (1:200 for IHC), and β-actin (1:5000 for WB).

### Co-immunoprecipitation

Mix the target protein with A/G magnetic beads and rotate on a shaker at 4 ^∘^C. Discard the culture medium from the cell culture dish and wash the cells three times with PBS. Add 1 mL of lysis solution and incubate for 10 min at room temperature. Scrape the cells using a cell scraper and divide the lysate into three EP tubes, labeling them as the Input group, IgG group, and protein group, respectively. Add loading buffer to the input group, heat at 100 ^∘^C for 10 min, and store at −20 ^∘^C. Place the remaining EP tubes on a magnetic stand, remove the supernatant, and add the appropriate antibodies to each group. Rotate at 4 ^∘^C overnight. The following day, collect the immunoprecipitates, wash them three times with IP lysis buffer, then add 15 µL of 2× loading buffer to each tube. Boil the samples for 10 min and proceed with Western blot detection.

### Ethical statement

This study was approved by the Ethics Committee of the Affiliated Haian Hospital of Nantong University. All patients provided written informed consent prior to their enrollment in the study.

### Statistical analysis

Statistical analyses were conducted using SPSS (version 20.0, IBM, Chicago, IL, USA), GraphPad Prism 7 (GraphPad Software, San Diego, CA, USA), and R (version 4.2.1, R Foundation for Statistical Computing, Vienna, Austria) with the ggplot2 package (version 3.4.4) for generating violin plots and heatmaps. Data normality was assessed using the Shapiro–Wilk test. Normally distributed data are presented as mean ± standard deviation (SD), while non-normally distributed data are reported as median with interquartile range (IQR). Group comparisons were performed using parametric tests (independent samples *t*-tests and paired *t*-tests) for normally distributed data, or non-parametric methods for non-normally distributed data. Survival curves were generated using Kaplan–Meier analysis, and differences between groups were assessed using the log-rank test. Multivariate Cox proportional hazards regression models were employed to calculate hazard ratios (HRs) with 95% confidence intervals (CIs) for survival outcomes. A *P* value less than 0.05 was considered statistically significant.

**Figure 1.**
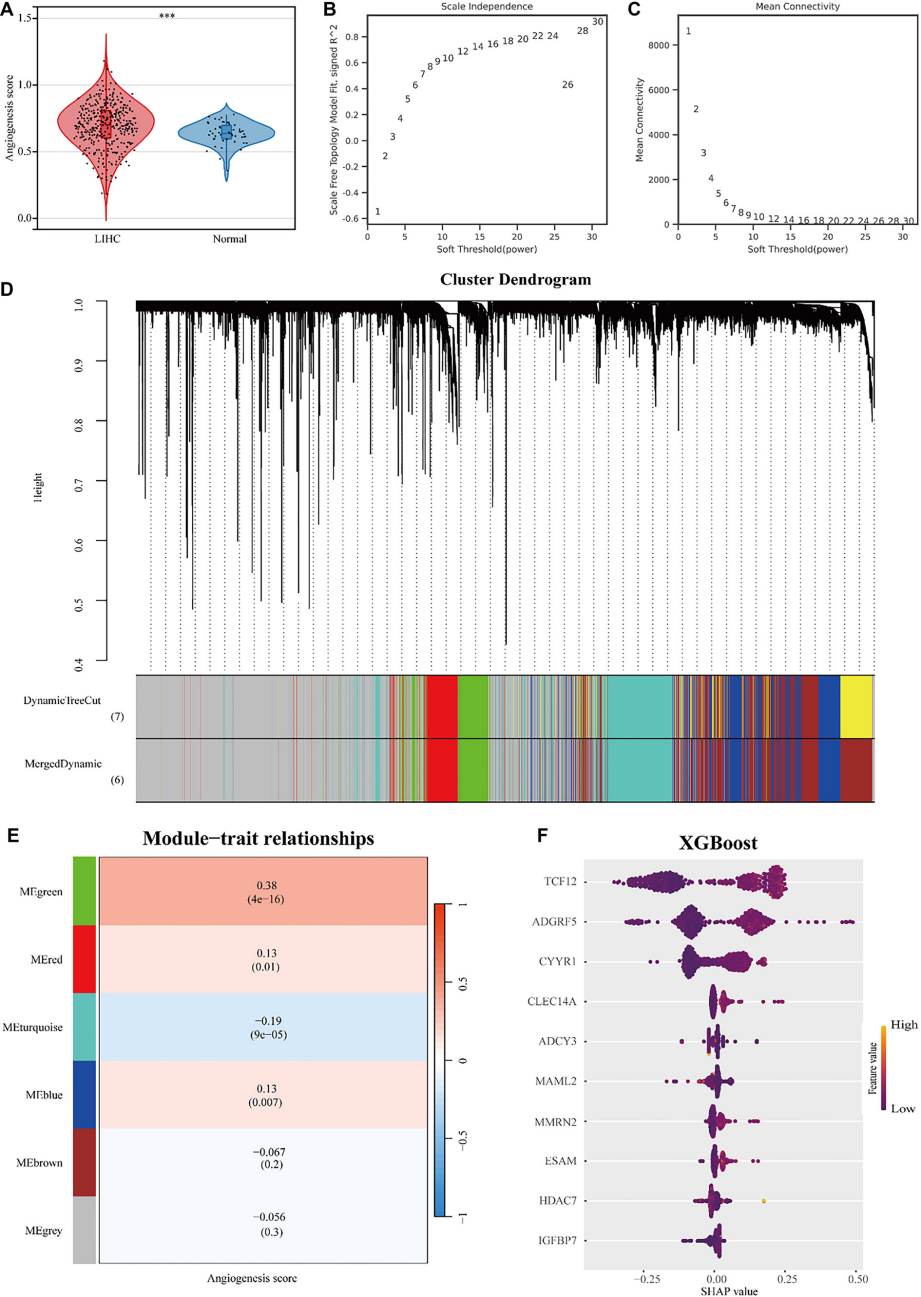
**TCF12 is a key regulatory gene for angiogenesis.** (A) Differences in expression of angiogenesis scores in liver cancer and normal liver samples; (B and C) WGCNA network construction parameters; (D) Cluster dendrogram; (E) Trait module association heat map; (F) Screening of key angiogenesis regulatory genes based on Xgboost algorithm. TCF12: Transcription factor 12.

## Results

### Machine learning identifies key regulatory genes for angiogenesis in liver cancer

The angiogenesis score for each sample in the TCGA-LIHC dataset was calculated using the ssGSEA method. Our results showed that the angiogenesis score in LIHC samples was significantly higher than in normal liver samples (Figure 1A). Gene co-expression network analysis was conducted to identify co-expressed gene modules and explore the relationship between these gene networks and angiogenic phenotypes. To ensure the network followed a scale-free distribution, it was necessary to select an appropriate value for the adjacency matrix weight parameter, referred to as “power.” We evaluated power values ranging from 1 to 30, calculating the corresponding scale-free topology fit index (correlation coefficient) and average connectivity for each. A higher correlation coefficient (maximum of one) suggests the network more closely approximates a scale-free topology; however, maintaining sufficient gene connectivity is also critical. Therefore, the selected power value needed to strike a balance between high connectivity and a high correlation coefficient. Based on this analysis, we selected a power value of 28 (Figure 1B and 1C). Using the selected power, we constructed a weighted gene co-expression network, classifying 14,928 genes into six distinct modules. Genes that could not be assigned to any specific module were grouped into the gray module (Figure 1D). Notably, genes in the green module showed the strongest correlation with the angiogenesis score (Figure 1E). Within the green module, we applied the XGBoost algorithm to rank genes based on their importance in predicting the angiogenesis score. This analysis identified TCF12 as a key regulatory gene involved in angiogenesis (Figure 1F).

**Figure 2.**
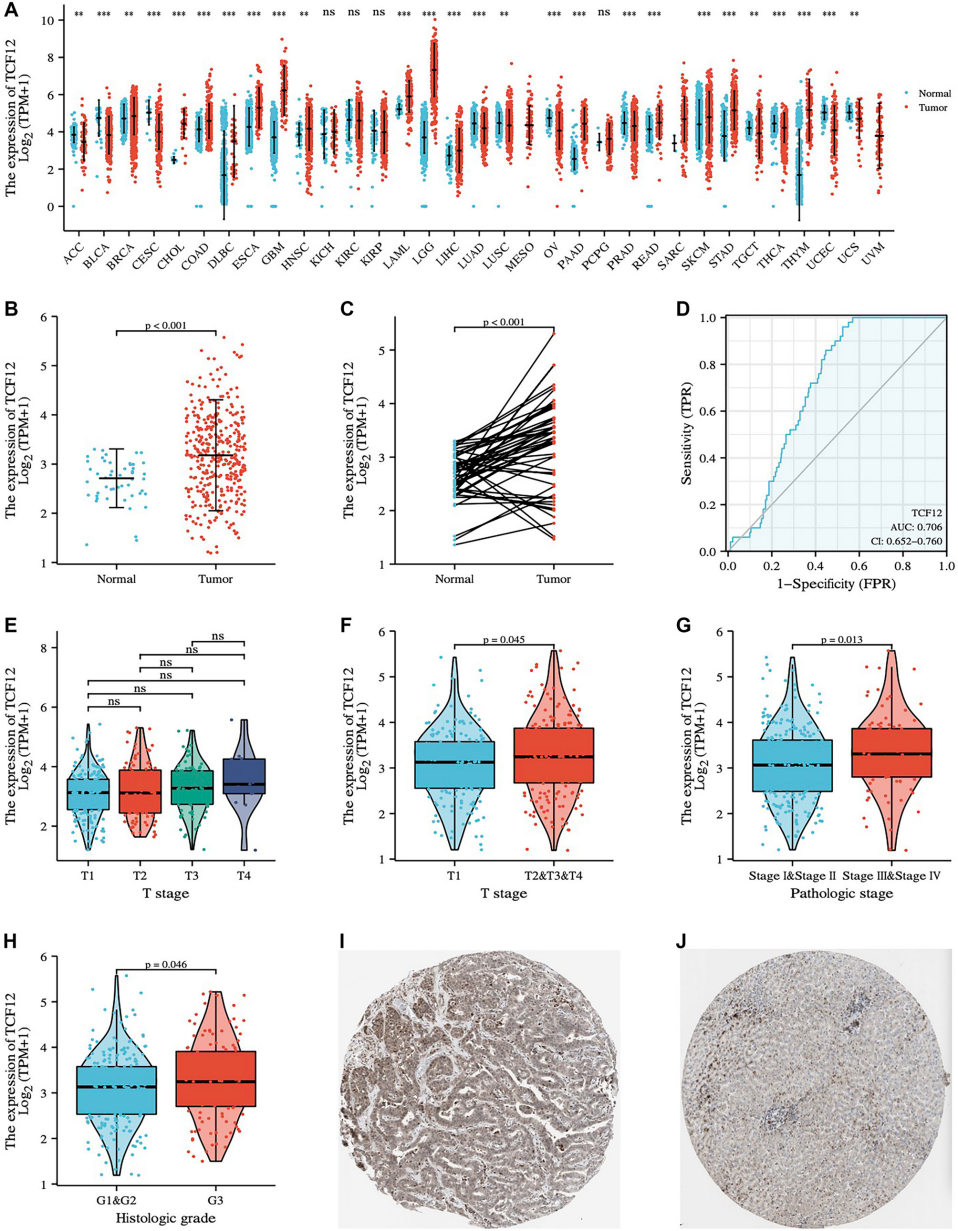
**Analysis of TCF12 in the database.** (A) TCF12 expression in pan-cancer; (B) Expression of TCF12 in unpaired liver cancer and normal liver tissue; (C) TCF12 expression in paired liver cancer and normal liver tissues; (D) ROC curve analysis of the predictive value of TCF12 on prognosis; (E) Expression differences among patients with T1, T2, T3, and T4 stages; (F) Expression differences between patients in T1 stage and T2+T3+T4 stage; (G) Expression differences between StageI+StageII and StageIII+StageIV patients; (H) Expression differences between patients in G1+G2 stage and G3 stage; (I and J) Expression of liver cancer and normal tissues in HPA database. TCF12: Transcription factor 12.

### Expression analysis of TCF12

The expression of TCF12 in cancer was initially analyzed using data from the TCGA database, which showed that TCF12 is generally upregulated across various cancer types (Figure 2A). In liver cancer specifically, TCF12 expression was significantly higher in 374 tumor samples compared to 50 normal liver tissues (Figure 2B). This trend was further confirmed in a cohort of 50 paired liver cancer and adjacent normal tissue samples, where TCF12 levels were also significantly elevated in cancerous tissues (Figure 2C). The prognostic significance of TCF12 in liver cancer was assessed using ROC analysis, yielding an AUC of 0.706. Patients were divided into high- and low-expression groups based on the optimal cutoff determined by the maximal Youden index (Figure 2D). TCF12 expression was also evaluated across tumor stage (T1–T4) and histologic grade (G1–G3). Due to limited sample sizes in some subgroups, stages T2–T4 were combined for analysis. Although no significant differences were observed among individual T stages (Figure 2E), combined T2–T4 tumors showed significantly higher TCF12 expression compared to T1 tumors (Figure 2F). Similarly, G3 (poorly differentiated) tumors had higher TCF12 levels than G1/G2 tumors (Figure 2H). Comparisons by overall pathologic stage (I–IV) are shown in Figure 2G. Finally, data from the Human Protein Atlas (https://www.proteinatlas.org/) confirmed that TCF12 expression is higher in liver cancer tissues compared to adjacent normal tissues (Figure 2I and 2J).

### Analysis of the correlation between TCF12 and prognosis of liver cancer patients

**Figure 3.**
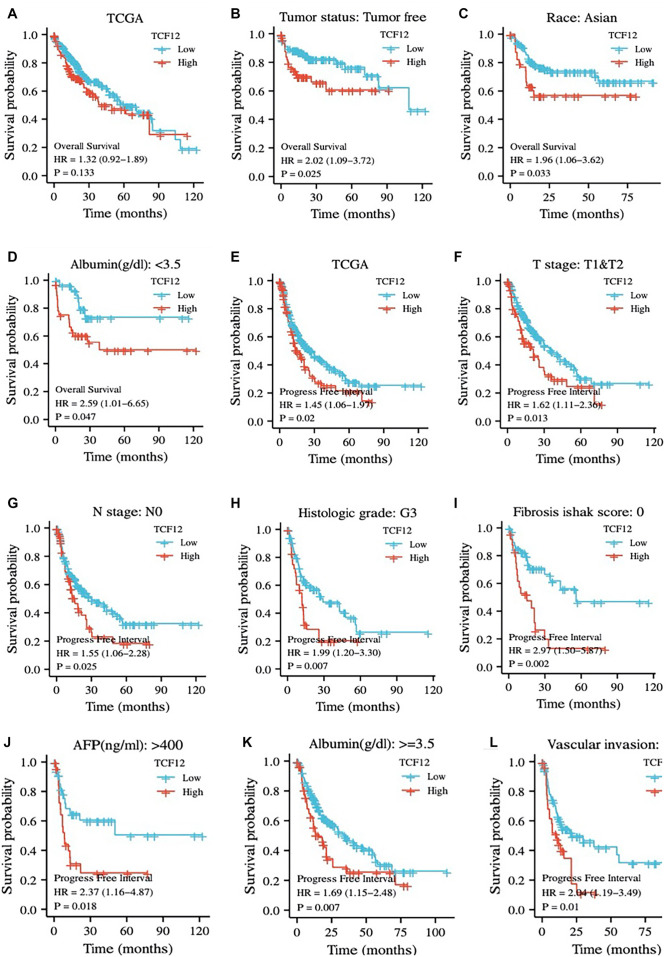
**Patients with high TCF12 expression have poor prognosis.** (A–D) Overall survival and (E–L) Progression-free interval. TCF12: Transcription factor 12.

To investigate the relationship between TCF12 expression and liver cancer prognosis, this study analyzed OS and progression-free interval (PFI) in various subgroups of liver cancer patients from the TCGA database. The results showed no significant difference in OS between patients with high and low TCF12 expression (Figure 3A). However, among tumor-free patients, Asian patients, and those with low plasma protein levels, high TCF12 expression was associated with significantly shorter OS compared to low expression (Figure 3A–3D). In addition, patients with high TCF12 expression had a significantly shorter PFI than those with low expression (Figure 3E). Furthermore, among patients at T1 and T2 stages, N0 stage, G3 grade, with an Ishak fibrosis score of 0, AFP >400 ng/mL, albumin >3.5 g/dL, and vascular invasion, those with high TCF12 expression showed reduced OS and PFI (Figure 3F–3L).

### TCF12 is highly expressed in liver cancer and is involved in regulating angiogenesis

**Figure 4.**
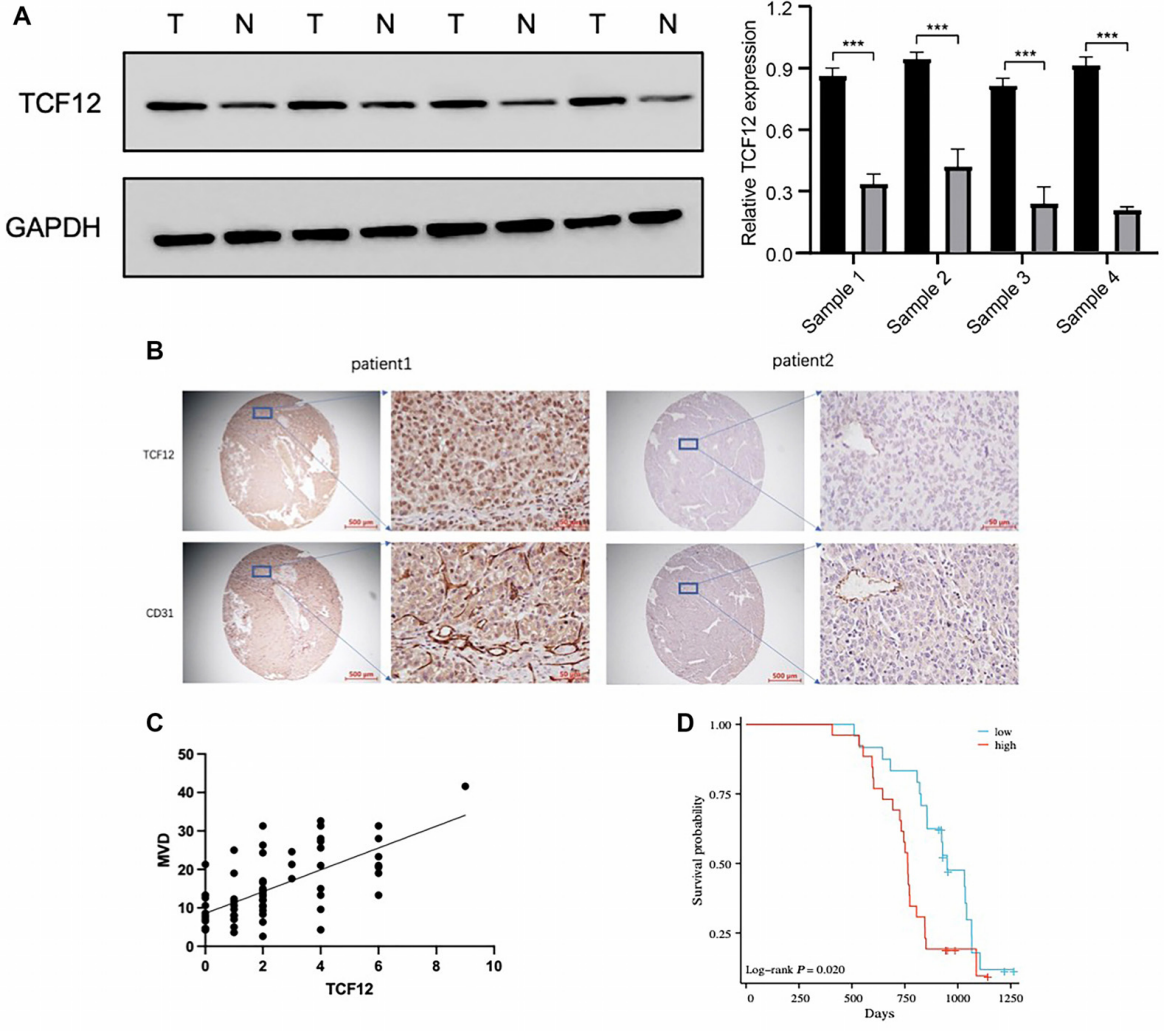
**TCF12 is highly expressed in liver cancer and related to blood vessel formation.** (A) Western blot detects the expression of TCF12 in four pairs of liver cancer and adjacent tissues; (B) Immunohistochemical detection of TCF12 and CD31 expression on liver cancer tissue chip; (C) Correlation between TCF12 expression and microvessel density; (D) Correlation between TCF12 expression and OS in liver cancer patients. TCF12: Transcription factor 12.

Initially, Western blot analysis was performed to compare TCF12 expression levels in liver cancer tissues vs adjacent non-cancerous tissues. The results showed that TCF12 protein levels were significantly higher in liver cancer tissues (Figure 4A). To further explore the relationship between TCF12 and angiogenesis, immunohistochemical staining was conducted on 50 liver cancer tissue samples to evaluate the expression of TCF12 and CD31. The analysis revealed a positive correlation between TCF12 and CD31 expression in liver cancer tissues (Figure 4B and 4C). Furthermore, patients with high TCF12 expression exhibited a lower OS rate (Figure 4D). These findings suggest that TCF12 is associated with angiogenesis and correlates with poor prognosis in liver cancer patients.

### TCF12 affects liver cancer angiogenesis and metastasis

**Figure 5.**
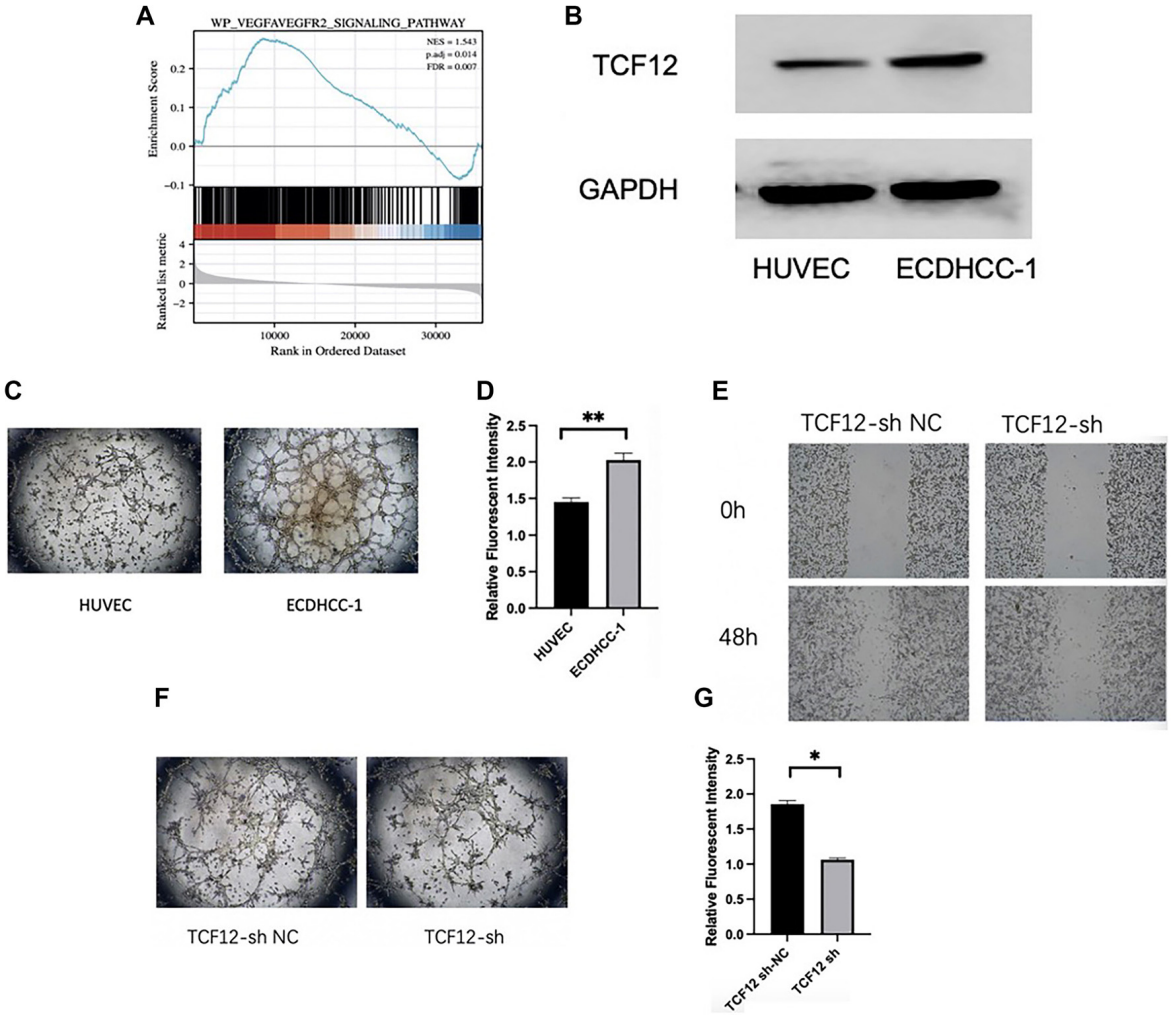
**TCF12 promotes blood vessel formation in liver cancer.** (A) TCF12 is now related to the VEGF signaling pathway; (B) Expression of TCF12 in HUVEC cells and ECDHCC-1 cells; (C) Comparison of tube-forming abilities of HUVEC cells and ECDHCC-1 cells; (D) Comparison of permeability of HUVEC cells and ECDHCC-1 cells; (E) Knockdown TCF12 scratch assay to detect migration ability; (F) Tube formation experiment by knocking down TCF12; (G) TCF12 knockdown permeability experiment. TCF12: Transcription factor 12; VEGF: Vascular endothelial growth factor; HUVEC: Human umbilical vein endothelial cell.

This study first identified a relationship between TCF12 and the VEGF signaling pathway through Gene Set Enrichment Analysis (GSEA) enrichment analysis (Figure 5A). Western blot analysis was then used to evaluate TCF12 expression levels in human umbilical vein EC (HUVEC) and ECDHCC-1 cells. Results showed that TCF12 protein levels were significantly higher in ECDHCC-1 cells compared to HUVEC cells (Figure 5B). Further comparison of tube formation and permeability between the two cell types revealed that ECDHCC-1 cells exhibited enhanced tube-forming ability and greater permeability (Figure 5C and 5D). Based on these findings, ECDHCC-1 cells were selected to investigate the functional role of TCF12. Lentiviral transfection was used to knock down TCF12 expression. Scratch assays demonstrated that TCF12 knockdown reduced the migratory capacity of ECDHCC-1 cells (Figure 5E), while tube formation assays confirmed that TCF12 knockdown impaired their ability to form tubes (Figure 5F). Additionally, permeability assays showed that TCF12 knockdown reduced cell permeability compared to the control group (Figure 5G). In summary, TCF12 promotes migration, tube formation, and permeability in liver cancer vascular ECs, suggesting it plays a key role in enhancing their angiogenic potential.

### Expression and prognostic analysis of HIF1A

**Figure 6.**
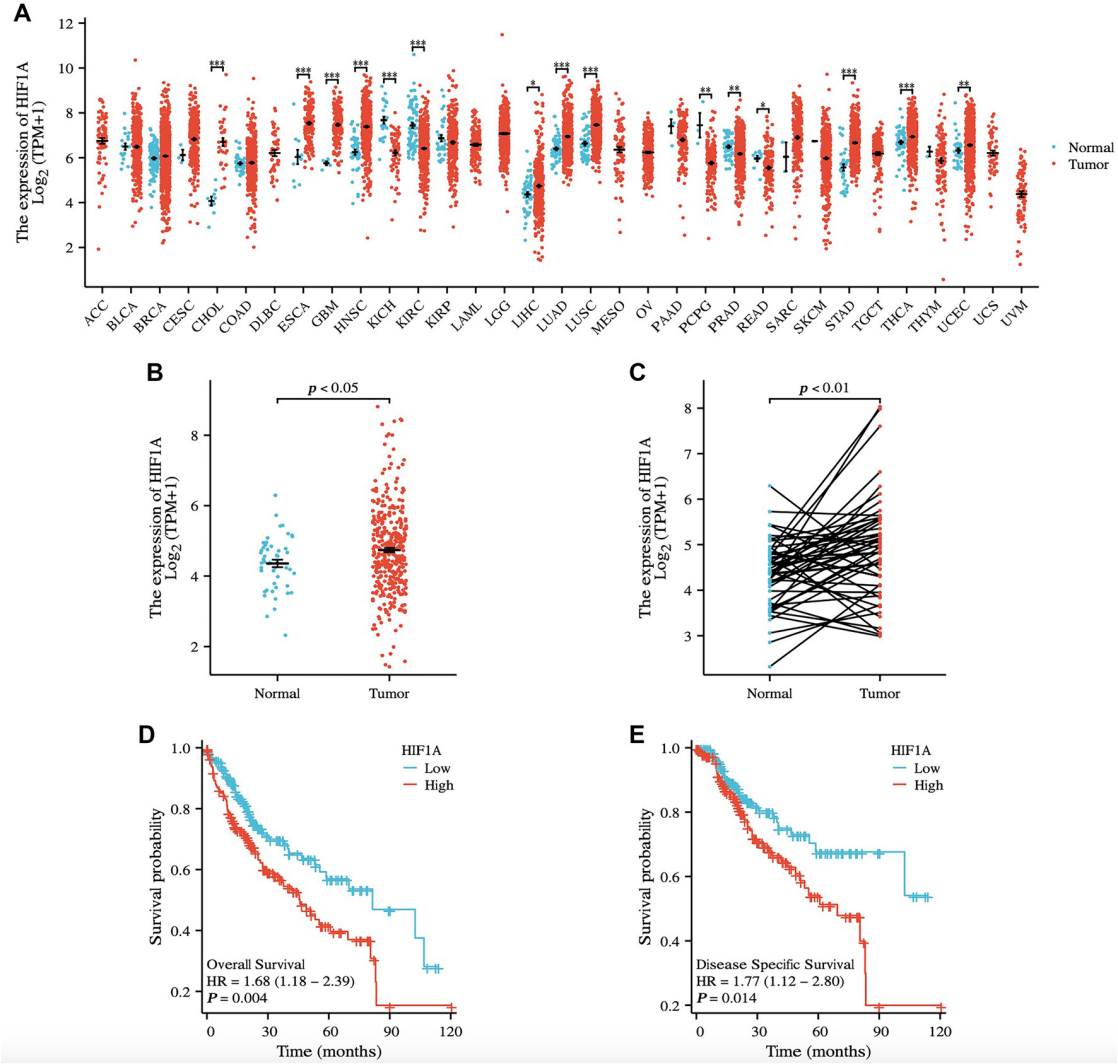
**Patients with high HIF1A expression have poor prognosis**. (A) HIF-1α expression in pan-cancer; (B) Expression of HIF-1α in unpaired liver cancer and normal liver tissue; (C) Expression of HIF-1α in paired liver cancer and normal liver tissues; (D) Relationship between HIF-1α expression and OS; (E) Relationship between HIF-1α expression and DSS. OS: Overall survival; HIF-1α: Hypoxia-inducible factor 1-alpha.

This study analyzed HIF-1α expression using data from the TCGA database. Results showed that HIF-1α levels were elevated in most tumors compared to normal tissues (Figure 6A). In liver cancer samples, unpaired tumor tissues exhibited significantly higher HIF-1α expression than adjacent normal tissues (Figure 6B and 6C). Additionally, patients with high HIF-1α expression had shorter OS and disease-specific survival (DSS) (Figure 6D and 6E).

### HIF-1 affects the migration and tube formation of liver cancer vascular ECs

**Figure 7.**
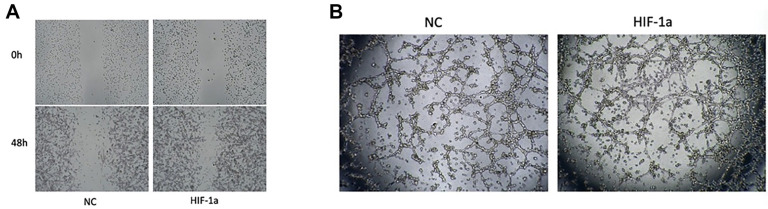
**Effect of HIF-1α on vascularization in liver cancer.** (A) Overexpression of HIF-1α promotes ECDHCC-1 cell migration and (B) Overexpression of HIF-1α promotes tube formation in ECDHCC-1 cells. HIF-1α: Hypoxia-inducible factor 1-alpha.

To investigate the effect of HIF-1α on the behavior of ECDHCC-1 cells, we first conducted a scratch assay, which revealed that overexpression of HIF-1α significantly enhanced the migratory capacity of the ECDHCC-1 cells (Figure 7A). Subsequently, we performed a tube formation assay, which demonstrated that HIF-1α overexpression also promoted the tube-forming ability of the ECDHCC-1 cells (Figure 7B).

### TCF12 interacts with HIF-1a

**Figure 8.**
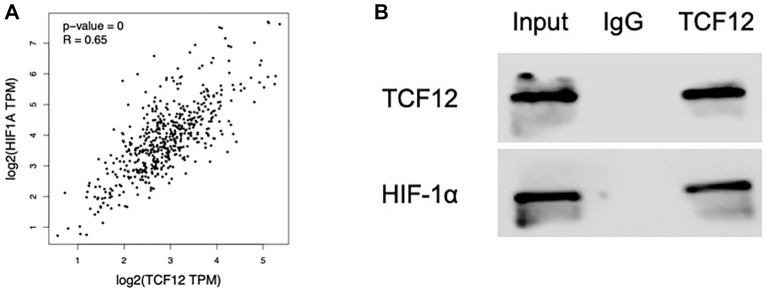
**TCF12 interacts with HIF-1α.** (A) Database analysis of the correlation between TCF12 and HIF-1α and (B) Co-immunoprecipitation experiment of TCF12 and HIF-1α. HIF-1α: Hypoxia-inducible factor 1-alpha; TCF12: Transcription factor 12.

Both TCF12 and HIF-1α contain bHLH domains and are capable of forming dimers with other E proteins to regulate gene expression. This suggests that TCF12 and HIF-1α may interact. To explore this potential interaction, a correlation analysis between TCF12 and HIF-1α was conducted using a database, revealing a positive correlation (Figure 8A). To further investigate this interaction, co-immunoprecipitation was performed in ECDHCC-1 cells to assess their interaction *in vivo*. The experimental results confirmed that TCF12 and HIF-1α do indeed interact *in vivo* (Figure 8B).

**Figure 9.**
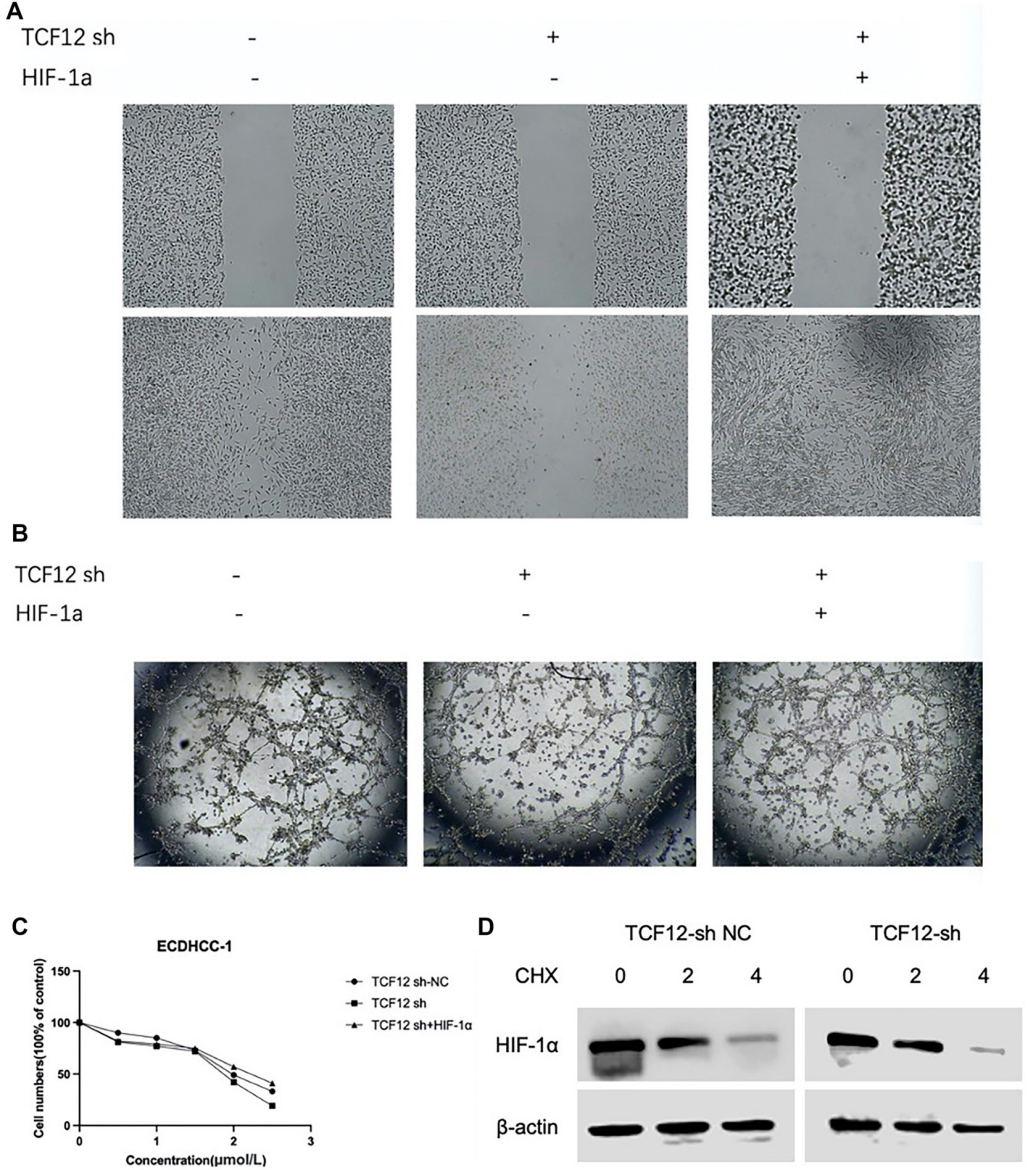
**TCF12 affects liver cancer vascularization and drug sensitivity by stabilizing HIF-1α.** Overexpression of HIF-1α reverses the (A) Weakened migration ability; (B) Tube-forming ability of ECDHCC-1 cells caused by TCF12 knockdown; (C) Enhanced sorafenib drug sensitivity; (D) TCF12 stabilizes HIF-1α expression. TCF12: Transcription factor 12; HIF-1α: Hypoxia-inducible factor 1-alpha.

### TCF12 affects vascularization and drug sensitivity of liver cancer by stabilizing HIF-1a

To investigate the effects of TCF12 and HIF-1α on tube formation and drug sensitivity in liver cancer vascular ECs, we examined three groups: a control group, a TCF12 shRNA group, and a TCF12 shRNA plus HIF-1α group. The results showed that TCF12 knockdown significantly inhibited ECDHCC-1 cell migration (by ∼50%) and tube formation (by ∼60%), while enhancing sorafenib sensitivity. Strikingly, HIF-1α overexpression partially reversed these effects, rescuing ∼80% of the migration capacity and ∼70% of the tube-forming ability (Figure 9A–9C). This study reveals the interaction mechanism between TCF12 and HIF-1α, demonstrating that TCF12 can stabilize HIF-1α expression (Figure 9D).

## Discussion

HCC is one of the most prevalent cancers and ranks as the second most lethal disease globally, with rising morbidity and mortality rates [18]. Targeted therapy plays a crucial role in the treatment of liver cancer [19]. However, due to drug resistance, most patients with advanced HCC are unable to achieve long-term benefits from systemic treatment, making HCC a highly fatal disease [20–22]. Therefore, understanding the mechanisms underlying liver cancer development and drug resistance is of significant importance. Sorafenib, a multi-kinase inhibitor [23], has been shown to inhibit tumor cell proliferation and exert anti-angiogenic effects [24]. It is currently one of the first-line treatments for liver cancer [25], and research into its treatment resistance has become a major area of focus. Angiogenesis is a complex and regulated process that occurs within the tumor microenvironment. ECs are stimulated by pro-angiogenic factors, leading to changes in their shape and migration toward the tumor. Simultaneously, ECs secrete matrix-degrading enzymes that break down the original vascular basement membrane. Following this, ECs proliferate, migrate, alter their permeability, and organize into a tightly connected luminal structure [4]. Throughout this process, EC migration is crucial, as it determines the direction of angiogenesis, facilitates the entry of new blood vessels into the tumor, and supplies essential nutrients and oxygen to tumor cells. Additionally, alterations in the tight junctions between cells during EC migration affect blood vessel permeability, facilitating the entry and exit of tumor cells into and from the bloodstream, which is critical for distant metastasis [26, 27]. Consequently, the regulation of EC migration and permeability is closely linked to intercellular tight junction proteins, playing a significant role in both tumor angiogenesis and drug resistance. It has been reported that TCF12 is involved in the occurrence and progression of various tumors [14–17]; however, few studies have investigated its role in liver cancer angiogenesis and drug sensitivity. This study first identified, through database analysis, that TCF12 is highly expressed in liver cancer, and that high TCF12 expression levels are associated with poor prognosis in liver cancer patients. GSEA revealed that TCF12 is linked to pathways associated with vascularization. Additionally, immunohistochemical staining performed on two sets of liver cancer tissue microarrays demonstrated a positive correlation between TCF12 expression and CD31 levels. These findings indicate that TCF12 is closely related to the vascularization of liver cancer. Furthermore, the analysis of TCF12 expression and prognosis in liver cancer patients suggests that those with high TCF12 levels have a worse prognosis. Collectively, these studies—integrating big data analysis and clinical specimen validation—demonstrate that TCF12 is significantly associated with angiogenesis. In exploring the biological characteristics of tumor vascular cells through functional assays, abnormal ECs are frequently generated by combining normal vascular ECs with genetic engineering techniques. However, the extent to which these cells accurately reflect the characteristics of tumor vascular ECs remains an area for further research. Ongoing concerns exist regarding the efficacy and specificity of vascular-targeted anticancer drugs previously evaluated using normal vascular EC models, especially their potential to destroy tumor blood vessels without causing toxicity to normal blood vessels. To address these differences, the research team developed immortalized human liver cancer vascular ECs. This study revealed that, compared to HUVEC cells, ECDHCC-1 cells exhibit higher protein levels of TCF12, enhanced tube-forming ability, and increased permeability. Additionally, when TCF12 was knocked down in ECDHCC-1 cells, a reduction in cell migration, tube formation, and permeability was observed. Angiogenesis is a hallmark of tumorigenesis, and HCC is characterized by a high density of blood vessels, which facilitates tumor development and progression [28]. Research has shown that under hypoxic conditions, HIF-1α transcriptionally activates the VEGF A VEGFA gene, promoting blood vessel formation in liver cancer [29]. Database analyses reveal that HIF-1α is highly expressed in liver cancer and correlates with poor prognosis in patients. Experimental studies on cell function have demonstrated that HIF-1α enhances the migration and tube-forming capabilities of liver cancer vascular ECs, as well as increases permeability between ECs. Co-immunoprecipitation experiments indicate that TCF12 interacts with HIF-1α, and additional studies suggest that TCF12 stabilizes HIF-1α. While our co-IP experiments confirm the TCF12-HIF-1α interaction, future studies should explore whether TCF12 directly modulates HIF-1α ubiquitination or recruits stabilizing cofactors (e.g., SIRT6 or p300). Proteomic analyses may also identify additional partners in this complex. Sorafenib has been shown to reduce blood vessel formation in tumors, leading to a hypoxic environment. Research indicates that intratumoral hypoxia in HCC contributes to resistance against sorafenib [30–32]. Enhancing sorafenib’s drug sensitivity is crucial for overcoming this resistance. This study investigated the effects of TCF12 and HIF-1α on tube formation and drug sensitivity in liver cancer vascular ECs. The knockdown of TCF12 resulted in increased sensitivity to sorafenib, while the overexpression of HIF-1α negated these effects. This study does have limitations. First, the downstream signaling pathways of HIF-1α remain underexplored. As a hypoxia-induced miRNA, miR-210 can establish a positive feedback loop with HIF-1α [33]. miR-210 is involved in various tumor processes and promotes cell proliferation while inhibiting apoptosis in lung cancer, breast cancer, and malignant glioma cells [34]. In HUVECs, miR-210 upregulation significantly enhances angiogenesis [35]. miR-210 can also activate the PI3K/AKT pathway by inhibiting ephrinA3, enhancing EC proliferation and migration [36]. It also targets PTP1B, negatively regulating it through the dephosphorylation of VEGFR2 in ECs. Additionally, VEGF signaling [37] disrupts the VE-cadherin and β-catenin complex, which weakens tight junctions between cells, leading to increased vascular permeability in liver cancer. Lastly, miR-210 targets the intercellular tight junction protein vacuole membrane protein 1 (VMP1) [38], reducing intercellular adhesion. The above research provides valuable insights into HIF-1α downstream pathways and holds significant reference value. Although this study used sorafenib-naive cells, the TCF12/HIF-1α axis may also contribute to acquired resistance. Future studies should explore whether TCF12 inhibition restores sorafenib sensitivity in resistant models and whether combining TCF12-targeted therapies with sorafenib enhances efficacy in advanced HCC. Future work will utilize orthotopic HCC mouse models to evaluate whether TCF12 knockdown synergizes with sorafenib to suppress tumor growth and angiogenesis. Such studies will strengthen the translational relevance of targeting the TCF12/HIF-1α axis. In conclusion, our study demonstrates that TCF12 promotes angiogenesis in liver cancer and influences the drug sensitivity of sorafenib. TCF12 may represent a promising new target for liver cancer treatment and could provide insights into addressing sorafenib resistance.

## Conclusion

The expression level of TCF12 in liver cancer was initially assessed using the TCGA database and Western blot analysis. Immunohistochemical staining was then performed on liver cancer tissue microarrays to investigate the correlation between TCF12 and CD31, as well as to evaluate the impact of TCF12 expression on the prognosis of liver cancer patients. Furthermore, several experiments—including scratch assays, tube formation assays, cell permeability assays, and cell viability assessments—were conducted to explore the effects of TCF12 and HIF-1α on vascularization and drug sensitivity in liver cancer. Lastly, the interaction between TCF12 and HIF-1α was confirmed through co-immunoprecipitation experiments.

## Data Availability

The datasets obtained from TCGA database (https://portal.gdc.cancer.gov/).
